# Association between health-related quality of life and heart rate variability in elderly individuals with cognitive impairment in Korea: cross-sectional study

**DOI:** 10.1186/s12877-023-04529-2

**Published:** 2023-12-13

**Authors:** Donghoon Kim, Jaeho Lee, Ju-Young Choi, Hyo-Jung Lee, Jin-Young Min, Kyoung-Bok Min

**Affiliations:** 1 Department of Preventive Medicine, Seoul National University College of Medicine, 103 Daehak-ro, Jongno-gu, Seoul, Republic of Korea; 2 Integrated Major in Innovative Medical Science, Seoul National University Graduate School, 103 Daehak-ro, Jongno-gu, Seoul, Republic of Korea; 3 Veterans Medical Research Institute, Veterans Health Service Medical Center, 53, Jinhwangdo-ro 61-gil, Gangdong-gu, Seoul, Republic of Korea

**Keywords:** Cognitive impairment, Heart rate variability, Quality of life, Korean

## Abstract

**Background:**

Cognitive impairment, a characteristic and prior stage of dementia, is a serious public health concern in Korea a country with rapidly aging population. In a neurovisceral integration model, cognitive ability is connected to emotional and autonomic regulation via an interconnection in the brain, which may be associated with health-related quality of life (HRQoL).

**Methods:**

This study investigated the association between the HRQoL and the autonomic nervous system (ANS) via EuroQoL-5 Dimension (EQ-5D) and heart rate variability (HRV) among 417 patients who visited the Neurology Department in Veterans Health Service Medical Center, Seoul, South Korea.

**Results:**

The mean age of 275 patients in the cognitive impairment group (CIG) was higher than that of 142 patients in the normal cognition group (NCG) (74.85 years vs. 72.96 years, *p* < 0.001). In a generalized linear model with a beta coefficient (β), an increase in HRQoL was associated with higher HRV levels was observed only in CIG (CIG: the standard deviation of all NN intervals (SDNN) (ln, ms): β = 0.02, *p* = 0.007; Total power spectral density (TP) (ln, ms^2^): β = 0.01, *p* = 0.007; High frequency (HF) (ln, ms^2^): β = 0.01, *p* = 0.015; Low frequency (LF) (ln, ms^2^): β = 0.01, *p* = 0.003) (NCG: SDNN (ln, ms): β = 0.01, *p* = 0.214; TP (ln, ms^2^): β = 0.01, *p* = 0.144; HF (ln, ms^2^): β = 0.00, *p* = 0.249; LF (ln, ms^2^): β = 0.01, *p* = 0.294).

**Conclusions:**

We found a significant association between HRQoL and HRV in Korean elders with cognitive impairment. However, this study is cross-sectional, so we cannot define direct causation. Further studies are needed to support our findings and to elucidate the biological mechanisms underlying these associations, especially in people cognitively impaired.

## Background

Cognitive impairment is an example of a neurodegenerative disease; thus, it is a serious public health problem in Korea, a country with a rapidly aging population [1–3]. Cognitive functions enable individuals to understand and interpret surrounding information, which is essential for everyday tasks and activities, and in complex situations [4]. Dysexecutive function, processing speed, working memory, sustained attention, behavioral inhibition, and general mental flexibility are cognitive functions associated with prefrontal cortex activity [5]. However, despite non-severe cognitive dysfunctions in individuals with cognitive impairment such as in dementia, health-related quality of life (HRQoL) may be negatively affected to some degree due to specific functional impairments. [6, 7].

Cognitive function often worsens under the dysfunction of the autonomic nervous system (ANS) [8]. The ANS generally consists of two major branches, the sympathetic system related to energy mobilization and the parasympathetic system related to vegetative and restorative functions [5]. The heart rate variability (HRV) analysis, which is a noninvasive examination that assesses ANS activity, includes variations between heartbeats of sympathetic and parasympathetic nerve system [9]. Higher HRV values represent better ANS function, whereas lower HRV values represent disease status and defective ANS function [9]. HRV can be used to predict total mortality, sudden death, cardiovascular disease risk, and other morbidities, and to measure physiological changes in psychiatric illnesses such as depression, anxiety disorder, panic disorder, and posttraumatic stress disorder (PTSD) [10, 11].

Numerous studies have emphasized that ANS activity has significantly impacted HRQoL patients with schizophrenia, end-stage renal disease, paroxysmal atrial fibrillation, and chronic obstructive pulmonary disease, since the altered ANS activity is a common physiological outcome of the diseases [12–16]. In the asymptomatic group consisting of those aged between 20 and 54 years, ANS activity has been associated with a decreased physical QoL because smoking, alcohol, and other cardiovascular risks may be related to physical condition [17]. Additionally, other studies suggest that sympathetic autonomic activity is associated with emotion regulation, such as controls of emotional stability and perseverative thinking, which are in turn associated with a dissatisfied life and lower QoL [18, 19].

Although previous studies have investigated a significant association between HRQoL and HRV depending on certain disease patients, there has been no study on people with cognitive impairment. In this study, we compared the association in Korean older adults with cognitive impairment with those with normal cognition.

## Methods

### Study population

This study recruited patients over 60 years old who received medical care at the Neurology Department in Veterans Health Service Medical Center, Seoul, South Korea in 2021–2022. The recruitment criteria were as follows: (1) patients who complained of cognitive decline; (2) patients capable of completing clinical tests and questionnaires by themselves; (3) patients who consented to participate in the study; (4) patients with no medical history of dementia (ICD-10: F00-F09, G30), brain infarction, cerebral hemorrhage, and Parkinson’s disease; and (5) patients with no medical history of fatal disease (e.g., cancer or mental illness). Criteria for inclusion were determined by expert neurological clinicians. The study protocols obtained approval from the Institutional Ethical Review Board of the Veterans Health Service Medical Center (IRB no. BOHUN 2021-02-024-001, BOHUN 2021-01-066-006).

**Fig. 1:**
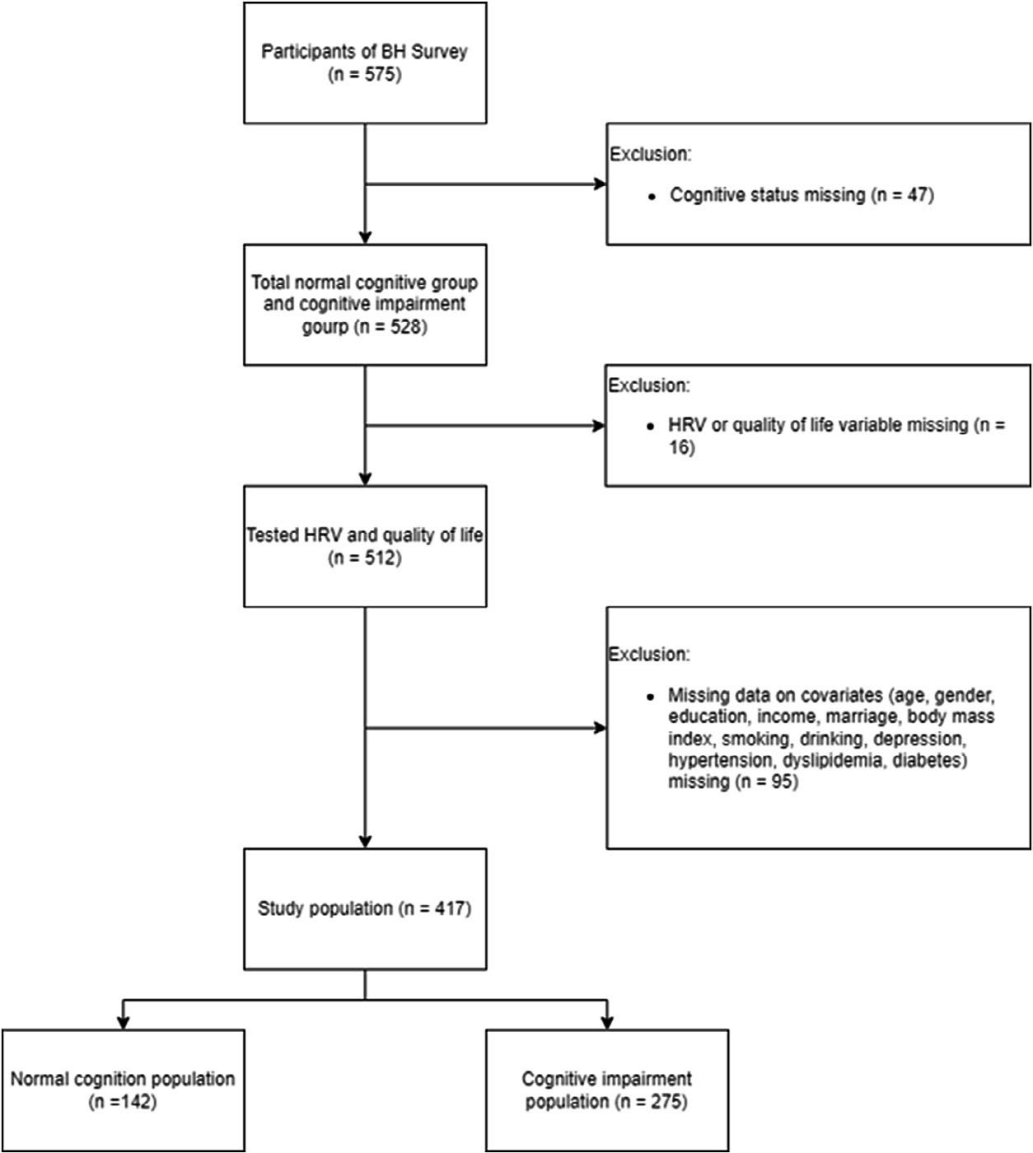
Selection of the study population

A total of 575 patients volunteered for the study and underwent a health survey, comprising HRV, cognitive examinations, and questionnaires, at the Veterans Medical Research Institute of the Veterans Health Service. Among these, 66 patients were excluded from the study due to missing cognitive examinations (*n* = 17), HRV (*n* = 13), and quality of life (*n* = 6) data; and/or diagnosed dementia (*n* = 30). The final study population included 417 patients with no missing data for the following covariates: age, sex, education, income, marital status, body mass index, smoking, drinking, depression, hypertension, dyslipidemia, and diabetes (*n* = 95) (Fig. 1).

A three-lead wireless electrocardiogram (ECG) recording device (MINDD SCAN, Ybrain Inc., South Korea) was used for HRV analysis. The recording was conducted in a quiet room for 5 min where the patients were in a rested sitting position, and electrodes were placed on the wrists and right ankle in a standard three-lead position. The patients were instructed to breathe normally and sit motionless without sleeping or talking during the recording. The time and frequency domain HRV analysis was performed using the Ybrain software (MINDD SCAN, Ybrain Inc., South Korea).

### Heart rate variability

The study used SDNN and percentage of pairs of adjacent intervals differing by more than 50 ms in the collection period (pnn50) among the time-domain indices. For the frequency-domain indices, LF (0.04–0.15 Hz, ms^2^), HF (0.15–0.40 Hz, ms^2^), total power spectral density (TP, ms^2^), and the LF/HF ratio were used. The pnn50 and the HF reflected parasympathetic activity, whereas LF represented both sympathetic and parasympathetic activity. The LF/HF power ratio reflected the sympathovagal balance, whereas SDNN and TP revealed the variability in the recording period which reflected global HRV [4, 9].

### Quality of life

The patients completed the Korean version of the EuroQoL-5 Dimension (EQ-5D) and visual analogue scale (VAS). The EQ-5D-3 L was developed by the European QOL group for measuring HRQOL and is divided into five dimensions: mobility, self-care, usual activities, pain/discomfort, and anxiety/depression. The patients evaluated each dimension as one of the following three levels: no problems, some or moderate problems, and extreme problems. Then, the total EQ-5D-3 L index was calculated by a Korean value set [20]. The VAS score ranged from 1 to 5 for self-health estimation. The EQ-5D-3 L and VAS provided a brief report on the quality of life of the participants as a single index of health status that can be applied for clinical and economic evaluation of health care and population health surveys.

### Neuropsychological battery test

The brief version of Seoul Neuropsychological Screening Battery (SNSB), named SNSB-Core (SNSB-C), was conducted to evaluate the level of core cognitive ability in the five cognitive domains: attention, language and related functions, visuospatial functions, memory, and frontal/executive functions [21]. The SNSB-C comprises 14 sub-tests derived from the SNSB: Vigilance Test, Digit Span Test, Comprehension Test, Repetition Test, short form of the Korean-Boston Naming Test, Ideomotor Apraxia Test (IAT), Rey Complex Figure Test, Seoul Verbal Learning Test-Elderly’s version (SVLT-E), Contrasting Program, Go-No Go Test, short form of the Korean-Color Word Stroop Test, Controlled Oral Word Association Test (COWAT), Korean-Trail Making Test-Elderly’s version (K-TMT-E), and Digit Symbol Coding [21].

For screening patients with cognitive impairment, the composite SNSB-C is an indicator of overall cognitive function and a substitute for the Korean Mini-Mental State Examination, a global instrument used to assess cognitive abilities briefly [21]. The effects of age, education years, and sex on the composite SNSB-C score were adjusted using the z-score standardization [21]. Patients were divided into two groups, the normal cognition group (NCG) and the cognitive impairment group (CIG). The patients were categorized under CIG if at least one of the percentiles in the sub-tests of the SNSB-C was less than 16th.

### Statistical analysis

To compare the statistical difference in participants’ characteristics between NCG and CIG, we performed the Chi-square test for categorical variables (i.e., sex, income, marital status, smoking, alcohol use, and history of disease) and the T-test for continuous variables (i.e., age, education year, and BMI). Since HRV variables are naturally non-normally distributed, the data were log-transformed to reduce skewness and reach normal distribution [22]. However, HRV data did not assume normality, so non-parametric methods were applied for the statistical analysis. Spearman rank correlation coefficients were used to examine the correlation between HRV and HRQoL, stratified by NCG and CIG. To assess the association between HRQoL and HRV by the presence of cognitive impairment, we conducted non-parametric regression by a multivariate generalized linear model. Each HRV variable was defined as an independent variable in the regression model, and HRQoL was described as a dependent variable. The model provided the beta coefficient (β) and 95% confidence interval (95% CI). The beta coefficient (β) is the regression coefficient and means the estimated effects or changes in the dependent variable (HRQoL) for a one-unit change in the independent variable (HRV). All regression models were adjusted for age, sex, education, monthly income, marital status, smoking, alcohol consumption, body mass index, depression, diabetes, and hypertension. Statistical analyses were performed using the Statistical Analysis System version 9.4 (SAS Institute, Cary, NC, United States), and the statistical significance level was set at p ≤ 0.05.

## Results

### Participant characteristics

Table 1 shows the characteristics of the population (n = 417) and the comparison of characteristics between NCG (n = 142) and CIG (n = 275). The characteristics expressed as mean in all populations were 74.21 years (age), 10.6 years (education), and 24.85 kg/m^2^ (BMI). The rest of the characteristics were expressed as proportions, 55.4% were male; 82.25% were married; 5.28% were smokers; 33.33% consumed alcohol; 32.13% had depression; 27.82% had diabetes; and 67.15% had hypertension. The mean age (72.96 vs. 74.85 years) and the proportion of those with diabetes (37.32 vs. 22.91%) were significantly different between the NCG and CIG.

**Table 1:** Characteristics of subjects in the NCG and CIG

	All study population Mean or N (95% CI or %) (n = 417)	NCG Mean or N (95% CI or %) (n = 142)	CIG Mean or N (95% CI or %) (n = 275)	*p*-value
Age (years)	74.21 (73.69 ~ 74.73)	72.96 (72.19 ~ 73.72)	74.85 (74.18 ~ 75.53)	0.005
Sex (Male, %)	231 (55.40%)	75 (52.82%)	156 (56.73%)	0.447
Education (years)	10.60 (10.17 ~ 11.03)	10.26 (9.53 ~ 11.00)	10.77 (10.24 ~ 11.30)	0.317
Monthly Income				
> 5 million (won)	43 (10.31%)	19 (13.38%)	24 (8.78%)	0.242
< 4 million (won)	88 (21.10%)	34 (23.94%)	54 (19.64%)	
< 3 million (won)	89 (21.34%)	26 (18.31%)	63 (22.91%)	
< 2 million (won)	106 (25.42%)	30 (21.13%)	76 (27.64%)	
< 1 million (won)	91 (21.82%)	33 (23.24%)	58 (21.09%)	
Marital status				
Married	343 (82.25%)	120 (84.51%)	223 (81.09%)	0.387
Divorced / Seperaated	74 (17.75%)	22 (15.49%)	52 (18.91%)	
Smoke status				
Current smoker	22 (5.28%)	5 (3.52%)	17 (6.18%)	0.47
Ex-smoker	144 (34.53%)	48 (33.8%)	96 (34.91%)	
Never smoked	251 (60.19%)	89 (62.68%)	162 (58.91%)	
Alcohol status				
Current drinker	139 (33.33%)	54 (38.03%)	85 (30.91%)	0.114
Ex-drinker	113 (27.10%)	30 (21.13%)	83 (30.18%)	
Never drunk	165 (39.57%)	58 (40.85%)	107 (38.91%)	
Body mass index (kg/m^2^)	24.85 (24.55 ~ 25.15)	24.99 (24.45 ~ 25.54)	24.78 (24.42 ~ 25.14)	0.578
Depression				
Yes	134 (32.13%)	37 (26.06%)	97 (35.27%)	0.056
No	283 (67.87%)	105 (73.94%)	178 (64.73%)	
Diabetes				
Yes	116 (27.82%)	53 (37.32%)	63 (22.91%)	0.002
No	301 (72.18%)	89 (62.68%)	212 (77.09%)	
Hypertesion				
Yes	280 (67.15%)	94 (66.2%)	186 (67.64%)	0.767
No	137 (32.85%)	48 (33.8%)	89 (32.36%)	

Table 2 shows the mean HRV and HRQoL in the study populations, NCG, and CIG. The mean HRV variables of the populations were 7.77 (SDNN, ln, ms), 0.11 (pnn50, %), 6.15 (TP, ln, ms^2^), 4.83 (HF, ln, ms^2^), 4.34 (LF, ln, ms^2^), and 0.97 (LF/HF, ln, ms^2^); the mean EQ-5D and VAS were 0.92 (EQ-5D) and 3.14 (VAS). The SDNN (7.79 vs. 7.75 ln, ms), TP (6.21 vs. 6.12 ln, ms^2^), HF (4.88 vs. 4.80 ln, ms^2^), LF (4.43 vs. 4.30 ln, ms^2^), LF/HF (– 0.45 vs. – 0.50 ln, ms^2^), and VAS (3.19 vs. 3.11) were higher in NCG than CIG. However, All the mean HRV and HRQoL were not significantly different between NCG and CIG.

**Table 2:** Mean HRV and QOL of subjects in the NCG and CIG

	All study population Mean (95% CI) (n = 417)	NCG mean (95% CI) (n = 142)	CIG mean (95% CI) (n = 275)	*p*-value
SDNN (ln, ms)	7.77 (7.69 ~ 7.84)	7.79 (7.66 ~ 7.92)	7.75 (7.66 ~ 7.84)	0.535
pnn50 (%)	0.11 (0.09 ~ 0.13)	0.10 (0.07 ~ 0.14)	0.12 (0.09 ~ 0.15)	0.892
TP (ln, ms^2^)	6.15 (6.00 ~ 6.30)	6.21 (5.95 ~ 6.48)	6.12 (5.93 ~ 6.30)	0.569
HF (ln, ms^2^)	4.83 (4.64 ~ 5.02)	4.88 (4.55 ~ 5.21)	4.80 (4.57 ~ 5.03)	0.503
LF (ln, ms^2^)	4.34 (4.17 ~ 4.52)	4.43 (4.14 ~ 4.71)	4.30 (4.08 ~ 4.52)	0.421
LF/HF (ln, ratio)	0.97 (0.88 ~ 1.07)	-0.45 (-0.64 ~ -0.27)	-0.50 (-0.63 ~ -0.37)	0.810
EQ-5D	0.92 (0.91 ~ 0.93)	0.92 (0.91 ~ 0.94)	0.92 (0.91 ~ 0.93)	0.458
VAS	3.14 (3.06 ~ 3.21)	3.19 (3.06 ~ 3.32)	3.11 (3.01 ~ 3.20)	0.291

Table 3 shows the correlation matrix between HRV and HRQoL in the NCG and CIG. In the NCG, the correlation coefficients were not significant. In contrast, the EQ-5D of the CIG, was significantly correlated with pnn50 (r = 0.13; *p* = 0.04), HF (r = 0.15; *p* = 0.01), and LF (r = 0.15; *p* = 0.01). The VAS was significantly correlated with SDNN (r = 0.17; *p* < 0.001), pnn50 (r = 0.18; *p* < 0.001), TP (r = 0.17; *p* < 0.001), HF (r = 0.18; *p* < 0.001), and LF (r = 0.21; *p* < 0.001). The range of significant correlation in the CIG was 0.13–0.15 for the EQ-5D and 0.17–0.21 for VAS.

**Table 3:** Spearman correlation of HRV and QOL in the NCG and CIG

	SDNN (ln, ms)		pnn50 (%)		TP (ln, ms2)		HF (ln, ms2)		LF (ln, ms2)		LF/HF (ln, ratio)	
	r	*p*	r	*p*	r	*p*	r	*p*	r	*p*	r	*p*
NCG (n = 142)												
EQ-5D	0.07	0.42	0.12	0.15	0.10	0.23	0.09	0.31	0.10	0.22	-0.07	0.43
VAS	0.03	0.75	0.03	0.69	0.03	0.76	0.01	0.91	0.03	0.69	-0.05	0.52
CIG (n = 275)												
EQ-5D	0.12	0.05	0.13	0.04	0.12	0.05	0.15	0.01	0.15	0.01	0.02	0.77
VAS	0.17	< 0.001*	0.18	< 0.001*	0.17	< 0.001*	0.18	< 0.001*	0.21	< 0.001*	-0.04	0.55

^*^ significant result after Bonferroni correction

Table 4 shows the beta coefficient and 95% confidencs intervals of the general linear model of HRQoL by HRV in the NCG and CIG. In the NCG, all linear models were not significant regardless of the adjustment, indicating no significant association between HRV and HRQoL. In contrast, EQ-5D was positively associated with SDNN (β = 0.02; *p* = 0.007), TP (β = 0.01; *p* = 0.007), HF (β = 0.01; *p* = 0.015), and LF (β = 0.01; *p* = 0.003). VAS was positively associated with SDNN (β = 0.18; *p* = 0.003), pnn50 (β = 0.72; *p* < 0.001), TP (β = 0.09; *p* = 0.004), HF (β = 0.08; *p* = 0.001), and LF (β = 0.07; *p* = 0.003) after adjustment.

**Table 4:** Generalized linear model of quality of life predicted in the NCG and CIG

	Unadjusted model				Adjusted model^*^			
	EQ-5D		VAS		EQ-5D		VAS	
	Beta coefficient (95% CI)	*p*	Beta coefficient (95% CI)	*p*	Beta coefficient (95% CI)	*p*	Beta coefficient (95% CI)	*p*
NCG (n = 142)								
SDNN (ln, ms)	0.01 (-0.01 ~ 0.04)	0.290	-0.01 (-0.19 ~ 0.16)	0.871	0.01 (-0.01 ~ 0.04)	0.214	0.02 (-0.15 ~ 0.19)	0.802
pnn50 (%)	0.02 (-0.06 ~ 0.11)	0.618	-0.04 (-0.68 ~ 0.61)	0.912	0.04 (-0.05 ~ 0.12)	0.395	0.22 (-0.43 ~ 0.87)	0.505
TP (ln, ms^2^)	0.01 (0.00 ~ 0.02)	0.240	-0.01 (-0.09 ~ 0.07)	0.795	0.01 (0.00 ~ 0.02)	0.144	0.01 (-0.08 ~ 0.09)	0.865
HF (ln, ms^2^)	0.00 (0.00 ~ 0.01)	0.271	-0.01 (-0.07 ~ 0.06)	0.818	0.00 (0.00 ~ 0.01)	0.249	0.01 (-0.06 ~ 0.08)	0.805
LF (ln, ms^2^)	0.00 (-0.01 ~ 0.01)	0.520	-0.03 (-0.11 ~ 0.05)	0.445	0.01 (0.00 ~ 0.01)	0.294	0.00 (-0.08 ~ 0.07)	0.922
LF/HF (ln, ratio)	-0.01 (-0.02 ~ 0.01)	0.321	-0.00 (-0.16 ~ 0.07)	0.449	0.00 (-0.02 ~ 0.01)	0.676	-0.04 (-0.16 ~ 0.08)	0.546
CIG (n = 275)								
SDNN (ln, ms)	0.02 (0.01 ~ 0.04)	0.005**	0.19 (0.07 ~ 0.31)	0.002**	0.02 (0.01 ~ 0.03)	0.007**	0.18 (0.06 ~ 0.29)	0.003**
pnn50 (%)	0.06 (0.01 ~ 0.11)	0.027	0.74 (0.34 ~ 1.14)	< 0.001**	0.04 (-0.01 ~ 0.09)	0.084	0.72 (0.33 ~ 1.10)	< 0.001**
TP (ln, ms^2^)	0.01 (0.00 ~ 0.02)	0.005**	0.10 (0.04 ~ 0.16)	0.002**	0.01 (0.00 ~ 0.02)	0.007**	0.09 (0.03 ~ 0.14)	0.004**
HF (ln, ms^2^)	0.01 (0.00 ~ 0.01)	0.005**	0.09 (0.04 ~ 0.13)	< 0.001**	0.01 (0.00 ~ 0.01)	0.015**	0.08 (0.03 ~ 0.12)	0.001**
LF (ln, ms^2^)	0.01 (0.00 ~ 0.02)	0.001**	0.09 (0.04 ~ 0.14)	0.001**	0.01 (0.00 ~ 0.01)	0.003**	0.07 (0.02 ~ 0.12)	0.003**
LF/HF (ln, ratio)	0.00 (-0.01 ~ 0.01)	0.711	-0.02 (-0.11 ~ 0.06)	0.576	0.00 (-0.01 ~ 0.01)	0.518	-0.03 (-0.12 ~ 0.05)	0.435

^*^Adjusted model for generalized linear model were adjusted for age, sex, education, monthly income, marriage, smoke, alcohol, body mass index, depression, diabetes, and hypertension

^**^Significant result after Bonferroni correction

**Fig. 2:**
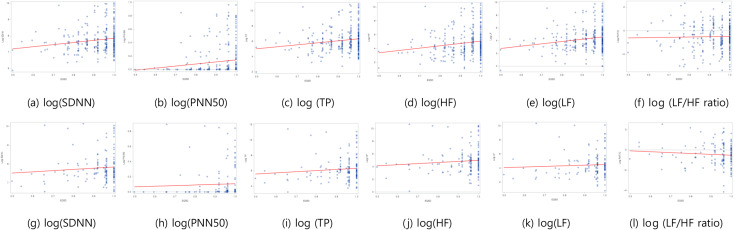
Plots of HRQoL and HRV in cognitive impairment group (**a-f**) and normal control group(**g-l**). The solid lines indicate regression lines

Figure 2 shows the regression lines and dot plotting of the association HRV and HRQoL in subjects with and without cognitive impairment.

## Discussion

We found that increased HRQoL was significantly associated with increased HRV variables in the CIG, whereas no association was found in the NCG. EQ-5D and VAS in the CIG, showed significant positive association with SDNN, TP, HF, and LF, and only VAS showed significant positive association with pNN50.

A recent nationwide population-based study in Taiwan investigated the relationship between HRQoL and cognitive impairment among 9,084 Taiwanese citizens aged over 65 years (normal cognition vs. MCI: 7035 vs. 2049) [7]. Those in the MCI showed having problem in all domains of HRQOL compared to normal individuals (beta coefficient (standard errors) in MCI with reference to normal group; mobility: β = 1.11 (0.09), *p* < 0.01; self-care: β = 1.58 (0.15), *p* < 0.01; usual activities: β = 1.35 (0.09), *p* < 0.01; pain/discomfort: β = 0.75 (0.06), *p* < 0.01; anxiety/depression: β = 0.76 (0.07), *p* < 0.01); overall HRQOL and VAS in MCI showed lower than normal (overall index of HRQoL: β = -0.16, *p* < 0.01; VAS: β = -6.68, *p* < 0.01) [7]. Another study in India suggested that the presence of low cognitive function reduced HRQoL by approximately 7% among older individuals as determined by the structural equation model approach [23]. We confirmed the relationship between HRQoL and cognitive impairment in older individuals through the previous studies.

Additionally, there have been studies investigating the association between HRV and cognition in older individuals. In 2018, the Geriatric Centre of the University of Campania Luigi Vanvitelli in Naples examined the HRV and cognitive ability of 117 older subjects (age > 65) for 24 h [10]. The study found an association between increased HRV and higher cognitive test scores from the Mini-Mental State Examination (MMSE; corrected for age and education) and the Montreal Cognitive Assessment (MoCA) test: the standard deviation of the average normal RR intervals over a 5-minute period (SDANN, ms) (β = 0.324, *p* < 0.05 for MMSE; β = 0.404, *p* < 0.001 for MoCA), SDNN (β = 0.390, *p* < 0.001 for MMSE; β = 0.481, *p* < 0.001 for MoCA), LF (β = 0.293, *p* < 0.001 for MMSE; β = 0.336, *p* < 0.001 for MoCA), and LF/HF (β = 0.293, *p* < 0.001 for MMSE; β = 0.326, *p* < 0.001 for MoCA) [10]. The result suggested an association between greater HRV parameters and better cognitive performance, especially parameters expressed sympathetic [10]. In contrast, another study targeted a cohort of women aged over 65 years and showed that the square root of the mean squared differences of successive normal RR intervals (RMSSD, ms), the number of interval differences of successive normal RR intervals greater than 50 ms (NN50, counts), HF power, and prevalent cognitive impairment according to MMSE [24]. Considering that the mechanism between autonomic dysfunction and cognitive impairment is unclear [4], these studies implied that both reduced sympathetic and parasympathetic functions may be associated with cognitive dysfunction.

Our results showed significant associations between HRQoL and HRV in patients with cognitive impairment. The prefrontal cortical areas, which include the orbitofrontal and medial prefrontal cortex, tonically inhibit the amygdala via GABAergic neurons in the amygdala [25, 26] Furthermore, prefrontal, cingulate, and insula cortices form an interconnected network with bidirectional communication with the amygdala [27]. The central nucleus of the amygdala (CeA) is the major efferent source to modulate the autonomic responses and, the disinhibition of CeA may lead to decreased HRV by lower activation of the prefrontal cortex which may lead to disinhibition of CeA. This in turn would disinhibit simultaneous sympathoexcitatory neurons in the rostral ventrolateral medulla (RVLM) and parasympathoexcitatory neurons in the nucleus of the solitary tract (NTS) [28].

The damage in the prefrontal cortex is also associated with the insula and cingulate due to communication. The insula is the main hub of the central autonomic network (CAN) and is implicated in both the regulation of ANS output and cognitive function via projections on preganglionic sympathetic and parasympathetic neurons [5]. Outputs of the CAN are under tonic inhibitory control via GABAergic neurons in the NTS [5, 8]. Emotional arousal is associated with concomitant decrease in brain activation in the right superior and dorsolateral prefrontal cortex and the left anterior cingulate cortex and a decrease in HRV [8]. The neurovisceral integration model considers the transmission of subcortical affective information to the cerebral cortex as required to experience emotion and that the top-down inhibitory influences related to the modulatory effect of subcortical shaped the nature of subjective experience [8]. Thus, prefrontal inhibitory control over brain activities and subcortical brain regions that are involved in emotional arousal and processing providing emotional dysregulation such as perseverative thoughts and emotional instability, which leads to life dissatisfaction and lower QoL [18]. Emotional dysregulation would be more serious among older individuals than the younger population because the prefrontal and amygdala activities are associated with age and cognition [29].

This study assumed that Korean olders with cognitive impairment might have ANS dysfunction and emotional dysregulation caused by problems in prefrontal inhibitory or brain activity. Therefore, this study suggested that impaired life satisfaction particularly related to health is associated with ANS among older individuals with cognitive impairment. To the best of our knowledge, the present study is the first study to report the association between HRQoL and HRV in a sample of older Korean individuals. The survey for EQ-5D, VAS, and HRV have benefits that include being non-invasive, relatively inexpensive, less time consuming, and does not have a complex procedure.

However, this study has a few limitations. First, since this study is based on a cross-sectional design, we cannot determine the causality for the observed association between HRV and HRQoL, especially in older adults who are cognitively impaired. Second, there are more powerful ways to monitor 24-hour Holter measurement than 5-min HRV measurement. However, short-term HRV remains stable and may be applicable for screening the variation in the heart rate. Third, cognitive functional status (herein, CIG vs. NCG) was assessed using a neurobehavioral test. Although the neurobehavioral examination is a clinical assessment of cognitive function, whether people are subject to cognitive impairment may be often misclassified when using brief cognitive assessments. Fourth, this study analyzed only specific HRV data (i.e., SDNN, TP, HF, and LF). Additional HRV data from Fourier transformation, approximate entry, and other measurements can be essential to clarify and assess the relationship between HRV and HRQoL. Finally, our study may be biased due to the limitation of unmeasured confounders and single-center study.

## Conclusion

We found a significant association between HRQoL and HRV in Korean elders with cognitive impairment. However, this study is cross-sectional, so we cannot define direct causation. Further studies are needed to support our findings and to elucidate the biological mechanisms underlying these associations, especially in people cognitively impaired.

## Data Availability

The datasets generated and analysed during the current study are not publicly available due to institutional restrictions but are available from the corresponding author on reasonable request.

## List of abbreviations

ANS: autonomic nervous system
CAN: central autonomic network
CeA: central nucleus of the amygdala
CIG: cognitive impairment group
EQ-5D: EuroQoL-5 Dimension
HF: High frequency
HRQoL: health-related quality of life
HRV: heart rate variability
LF: Low frequency
MMSE: Mini-Mental State Examination
MoCA: Montreal Cognitive Assessment
NCG: normal cognition group
NN50: the number of interval differences of successive normal RR intervals greater than 50 ms
NTS: nucleus of the solitary tract
pnn50: percentage of pairs of adjacent intervals differing by more than 50 ms in the collection period
RMSSD: square root of the mean squared differences of successive normal RR intervals
RVLM: rostral ventrolateral medulla
SDANN: standard deviation of the average normal RR intervals over a 5-minute period
SDNN: standard deviation of all NN intervals
TP: Total power spectral density
